# Metallocene Based Polyolefin Nanocomposites

**DOI:** 10.3390/ma7031995

**Published:** 2014-03-10

**Authors:** Walter Kaminsky

**Affiliations:** Institute for Technical and Macromolecular Chemistry, University of Hamburg; Bundesstr. 45, 20146 Hamburg, Germany; E-Mail: kaminsky@chemie.uni-hamburg.de; Tel.: +49-404-2838-3166; Fax: +49-404-2838-6008

**Keywords:** polyolefin nanocomposites, metallocene catalysts, inorganic particles, silica balls, carbon nanotubes, polyethylene, polypropylene

## Abstract

One of the most efficient and versatile ways to synthesize polyolefin nanocomposites is the *in-situ* polymerization of olefins in the presence of nano particles by metallocene catalysts. Metallocene/methylaluminoxane (MAO) catalysts are soluble in hydrocarbons and therefore they can be absorbed perfectly in solution onto the surface of particles or fibers and after addition of ethene or propene they can then catalyze a polyolefin film on the surface. Metallocene/MAO and other single site catalysts allow the synthesis of polymers with a precisely defined microstructure, tacticity, and stereoregularity as well as new copolymers with superior properties such as film clarity, high tensile strength and lower content of extractables. The polymer properties can be enlarged by the incorporation of nanofillers. The resulting polyethylene or polypropylene nanocomposites give a tremendous boost to the physical and chemical properties such as dramatically improved stiffness, high gas barrier properties, significant flame retardancy, and high crystallization rates.

## 1. Introduction

Within recent years, much research in academia and industrial laboratories has focused on the field of polyolefin nanocomposites because of their high potential as materials with novel properties such as improved mechanical properties stiffness, strength, toughness, increased heat distortion temperature, reduced permeability, and flame retardancy. An overview can be found in some books and review articles [1–4]. It was expected that these polymers would be the next materials after Ziegler-Natta and metallocene polyolefins [5]. The properties of the nanocomposites are not only influenced by the kind of fillers but also by the microstructure of the polyolefins and the preparation process. Metal powders, metal oxides, carbonates, silica, talcum, layered silica as well as fibers such as carbon nanotubes, carbon nanofibers, and polymer fibers are used as fillers [6–8]. The use of layered inorganic materials such as multilayered silicates, clay, montmorillonite, or graphene is of special interest, because of the additional great barrier resistance against permeation of gases and liquids [9–12].

Till today most composites are prepared by mechanical blending of the particles or fibers above the melting temperature of the desired matrix [13–15]. Melt compounding of polyolefins with nanoparticles often leads to an insufficient filler dispersion, especially at a high filler content, which leads to aggregation and intercalation, which in turn deteriorates the mechanical properties. Such disadvantages can be solved by *in-situ* polymerization whereby the cocatalyst methylaluminoxane (MAO) can be adsorbed or anchored on the surface of the nanofillers such as particles, fibers, carbon nanofiber (CNF), multi-walled carbon nanotubes (MWCNT), thereby changing the surface to a hydrophobic one [16,17]. An efficient technique to immobilize the MAO at the surface of the filler is the “polymerization-filling technique” (PFT) [18,19].

**Figure 1.**
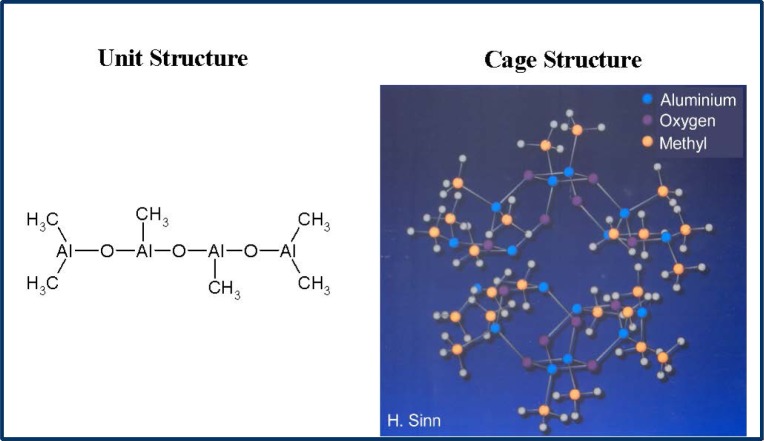
The main unit structure of methylaluminoxane (MAO) and the possible formation of a cage structure.

MAO is prepared by careful hydrolysis of trimethyl aluminium with water and is a mixture of different oligomers (Figure 1).The main oligomer unit contains four aluminium and three oxygen atoms as well as six methyl groups and forms bulky cages [20–22].

**Figure 2.**
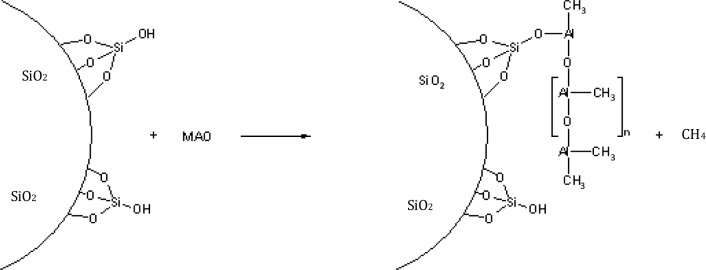
Hydroxyl or carboxyl groups present on the filler surface can react with added MAO to form a heterogeneous cocatalyst. The MAO is now anchored, but still able to form an active complex with the metallocene.

The MAO reacts, for example, with the OH-groups of silica or with carboxy groups of oxidized carbon nanotubes or is physically absorbed at the surface (Figure 2). Methane is formed by the chemical reaction of MAO with reactive polar groups.

Excess MAO is washed out. In a second step, the metallocene with the desired properties is added, forming catalytically active polymerization sites on the nanosurface. The thickness of the polymer films, formed by addition of ethene or propene, depends on the polymerization conditions, especially the polymerization time, the kind of metallocene catalyst, and the pressure of the monomer. The *in-situ* polymerization leads to composite materials where each particle or fiber is intensively covered with the polymer.

Metallocene/methylaluminoxane (MAO) and other single site catalysts are soluble in hydrocarbons, therefore they can be absorbed perfectly on the surface of particles and fibers or they can penetrate the layers of layered silicates and oxides [23,24]. Zirconocene complexes and half sandwich titanium complexes especially, have opened up a frontier in the area of new polymer synthesis and processing.

The transition metal complexes can be activated by MAO but also by other bulky cocatalysts such as perfluorophenylborate. By changing the ligand structure, these catalysts allow the synthesis of polymers with a tailored microstructure tacticity and stereoregularity as well as new copolymers with superior properties such as film clarity, tensile strength and lower extractables [25–30].

These single site catalysts give by *in-situ* polymerization of nanofillers a tremendous boost to the physical and chemical properties of polymers such as a dramatically improved stiffness with a neglible loss of impact strength, high gas barrier properties, significant flame retardancy, better clarity and gloss and high crystallization rates.

Heterogeneous nanosized Ziegler-Natta catalysts can also be used for *in-situ* polymerization of layered materials [18,31].

The composite materials show, for example, improved stiffness with negligible loss of impact strength, high gas barrier properties, significant flame retardancy, better clarity, and gloss as well as high crystallization rates. Even low nanoparticle contents are already sufficient to obtain new or modified material characteristics, particularly a faster crystallization rate and a higher crystallization temperature.

Especially polyethylene and isotactic or syndiotactic polypropylene nanocomposites have been investigated and required as materials for electronic, magnetic, and domestic devices and automotive applications with outstanding properties. Carbon nanofibers (CNF) or multiwalled carbon nanotubes (MWCNT) are an especially attractive class of fillers for polymers because of their intriguing mechanical and thermal properties [1,7,8,32]. It was a goal of our investigations to use inorganic particles, especially silica balls for polyolefin nanocomposites and carbon nanotubes to increase the stiffness, formstability and other properties.

## 2. Results and Discussion

We investigated the properties of polyethylene and polypropylene composites by the *in-situ* polymerization technique using different inorganic nano materials such as silica balls (monopheres) with a diameter of 200–250 nm, magnesium oxide, aluminium oxide, other inorganic materials as well as carbon nanotubes or fibers. Purified and oxidized nanotubes also bear functional groups such as hydroxyl or carboxyl on their surfaces. These groups react with MAO by the formation of a covalent oxygen aluminum bond, without deactivating effects for the catalysts. The MAO is now anchored, but still able to form a catalytically active complex with the metallocene.

**Figure 3.**
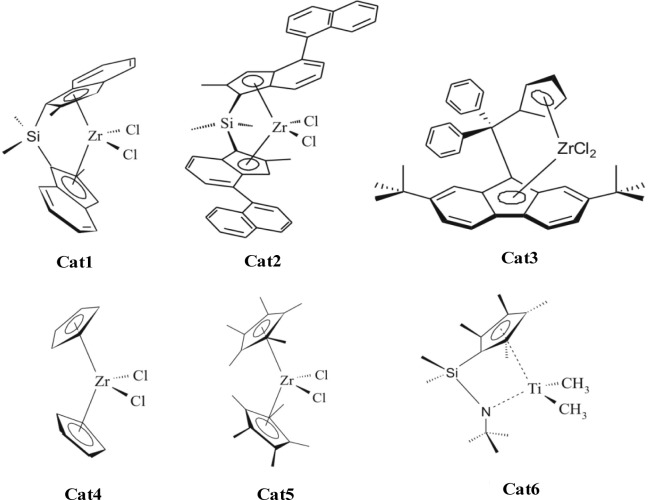
Structures of metallocenes used for the synthesis of polypropylene and polyethylene.

Figure 3 shows the metallocenes which are used by addition of MAO and ethene or propene to form the polyolefin nano composites. Cat1 and Cat2 produce isotactic polypropylene, Cat3 syndiotactic polypropylene, Cat4 atactic polypropylene. All shown metallocenes are able to synthesize polyethylene.

### 2.1. Polyolefin Nanoparticle Composites

The incorporation of nanoparticles caused an expansion of the application area, and particularly led to an improvement of the stiffness and crystallization velocity. Nanoparticles such as silica (monospheres, 200–250 nm), alumina (20 nm), magnesia (100 nm), calcium carbonate (70 nm), titanium carbide (25 nm), silicium carbide (25 nm), cadmium selenide (10 nm) boron nitride (200 nm) were used in the experiments. Selected ethene polymerization results are shown in Table 1 [33]. The catalyst was Cat1 [Me_2_Si(2-Me-Ind)_2_]ZrCl_2_ and MAO. The nanocomposites were prepared with metallocene catalysts by a slurry and gas phase reaction.

The polymerization activity is high and reaches 243,000 kg_PE_/mol_Zr_ in 1 hour. The molecular mass is nearly independent of the kind of nanoparticles of the same filler content. Activity and molecular weight (Mw) decrease hardly if the filler content increases from 1.5% to 9.6% in the case of cadmium selenide. These composite materials are of interest for the electronic industries, because a polyethylene film of a high electric resistance covers every nano selenide particle.

The results are similar, if polypropylene nanocomposites are produced using Cat2 and propene (Table 2). The activities and the molecular weights of the produced polypropylene differ by the kind of filler and with the filler content. Again, a higher filler content shows a lower activity.

For the preparation of syndiotactic polypropylene nano-silica-composites Cat3 [(pMePh)_2_C(Cp)(2,7-bis-tBuFlu)]ZrCl_2_ was used [34]. Compared to the isotactic polymerization the activity is less. Therefore the amount of MAO was optimized and triisobutyl aluminium (TIBA) used as scavenger (Table 3).

To find the minimum amount of MAO needed to activate the metallocene (Cat3) and in order to determine whether the applied amount of MAO had any influence on the envelopment of the silica spheres, different amounts of MAO were applied to the silica spheres for heterogenization.

All other conditions (solvent volume, polymerization temperature, amount of M200/MAO, polymerization time, catalyst amount) were kept constant. Several polymerizations were performed with each batch of M200/MAO to determine the optimum amount of MAO for the impregnation. The maximum activity of 3400 kg_Pol_/(mol_Zr_·h·mol_Mon_/L) was reached when a MAO amount of 420 mg/g M200 was used for the impregnation. For higher amounts of 830 mg MAO/g M200, the activity of the corresponding catalysts decreased to 2400 kg_Pol_/(mol_Zr_·h·mol_Mon_/L), respectively. All detected melting temperatures lay in the region of 140 °C with no distinct trend visible regarding the applied amount of MAO. The same is true for the molar masses ranging from 500,000 to 600,000 g/mol.

**Figure 4.**
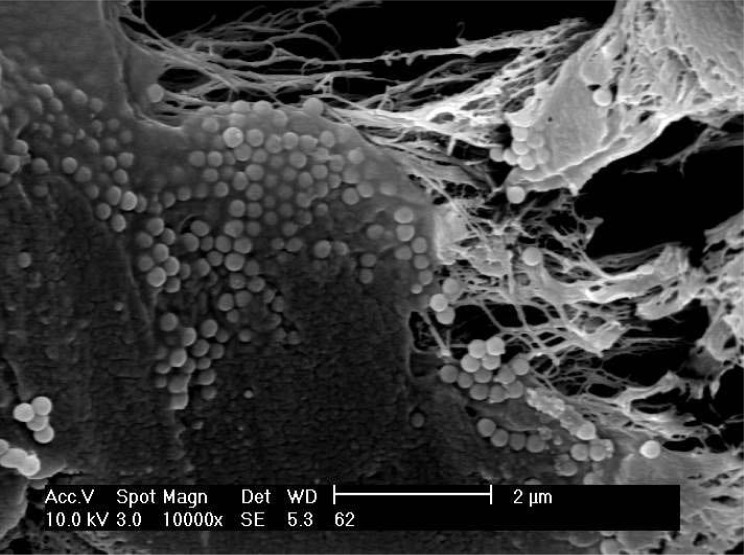
SEM micrograph of sPP/M200 nanocomposite (magnification 10,000×), prepared by 60 °C (see Table 3).

The coverage of the silica spheres with syndiotactic polypropylene is shown in Figure 4. Every monosphere is covered with a thin film of 20–50 nm of sPP. There are some polypropylene fibers which contain less silica balls due to different active sites. An amount of 420 mg MAO was used for 1 g of M200. The content of fillers in the sample is around 40 wt% and the rrrr pentade around 91%.

To reach a better dispersion and envelopment of the silica spheres with polypropylene, the influence of the polymerization temperature was investigated. To ensure comparability among the results, one batch of M200/MAO was synthesized as described above and used as cocatalyst for all subsequent slurry polymerizations. In Table 3 the temperature dependence of the *in-situ* polymerization of silica and propene at 0, 30 and 60 °C is listed. A higher polymerization temperature expectedly included a higher activity. The average activity of the polymerizations with M200/MAO/1 as cocatalyst was much lower than that of comparable homogeneous polymerizations with MAO/1. For 0 °C it was only 600 kg_Pol_/(mol_Zr_·h·mol_Mon_/L) with the heterogeneous system M200/MAO/3 as compared to 2000 kg_Pol_/(mol_Zr_·h·mol_Mon_/L) for the homogeneous polymerization.

At 30 °C it was 2300 compared to 5200 kg_Pol_/(mol_Zr_·h·mol_Mon_/L) and at 60 °C it amounted to 2400 compared to 9500 kg_Pol_/(mol_Zr_·h·mol_Mon_/L). As expected, the highest melting temperature of 149 °C was reached for polypropylenes synthesized at 0 °C, then decreased to 141 °C at 30 °C polymerization temperature and to 121 °C at a polymerization temperature of 60 °C, corresponding to a sinking syndiotacticity. The concentration of propene in the reaction mixture was varied in the range from 0.6 to 3.5 mol/L. According to Table 1, hardly any dependence was found of the activity, lying between 1800 and 2500 kg_Pol_/(mol_Zr_·h·mol_Mon_/L), on the concentration. Only at a propene concentration as low as 0.6 mol/L, did the average activity sink to 1800 kg_Pol_/(mol_Zr_·h·mol_Mon_). Likewise, the melting point of approximately 137 °C lay about 4 °C below that of the polypropylene from the slurry polymerizations at 1.4 mol/L propylene at otherwise the same conditions. For the highest propylene concentration of 3.5 mol/L, the melting temperature lay in the same range as for polymers produced at a concentration of 1.4 mol propylene per liter. The molecular weight was naturally influenced by the monomer concentration and increased from 370,000 to 640,000 g/mol for propylene concentrations of 0.6–3.5 mol/L, respectively.

**Figure 5.**
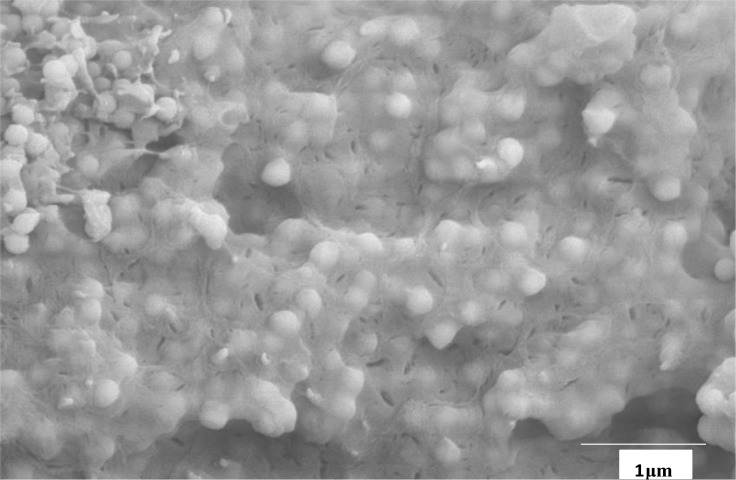
SEM micrograph of silica (monospheres) in an isotactic polypropylene matrix prepared by *in-situ* polymerization in the gas phase with rac-[Et-(IndH_4_)_2_]ZrCl_2_/MAO as catalyst filler content 50 wt%, magnification 10,000×.

In the gas phase, experiments were carried out using rac-[Et-(IndH_4_)_2_]ZrCl_2_ which produces isotactic polypropylene. The polymerization was carried out at 30 °C for 60 min with a metallocene amount of 6.25 × 10^−6^ mol. The propylene concentration was 0.2 mol/L and 0.55 g M200/MAO prepared as described above were used as cocatalyst. An activity of 400 kg_Pol_/(mol_Zr_·h·mol_Mon_/L) could be reached. Figure 5 shows an isotactic polypropylene/silica composite material with 50 wt% of monospheres. There is no agglomeration of the silica balls. The composite material has properties between polymer and ceramic. The melting temperature of the isotactic polypropylene is 138 °C. The thickness of the polypropylene layer covering the monospheres lies approximately between 20 and 100 nm.

To determine whether MAO-impregnated silica balls are suitable for storage 2.55 g silica balls were impregnated with 11 mL of MAO-solution (10% MAO). Polymerizations of propene were carried out at 30 °C directly after preparation, 2 weeks later, and 11 weeks later. As can be seen from Table 4, the activities decreased only slightly even after 11 weeks to 81% of the original activity. This shows that nanofiller/MAO precursors can be stored over a long period [35].

In order to generate the active complex from the zirconocene and the bonded MAO, the metallocene has necessarily to diffuse to the heterogeneous cocatalyst. Thereby the polymerization takes place near to the filler surface (see Figure 2). This leads to a polymer growth directly on the nanoparticle, results in excellent filler coverage with polymer, and promotes the separation of the individual particles during the polymerization. Furthermore, the hydrophobic character of some filler materials such as carbon nanotubes supports the drawing onto the fiber.

### 2.2. Polyolefin Carbon Nanocomposites

Carbon nanofibers (CNF) and carbon nanotubes (CNT) are an especially attractive class of fillers for polymers because of their intriguing mechanical and thermal properties. They represent one of the strongest and toughest materials known. An entire separation, homogeneous distribution and a good adhesion of the polymer matrix and the single- or multi-walled carbon nanotubes (SWCNT, MWCNT) are important to achieve the full potential of the resulting PE/CNT nanocomposite. The pre-treatments and PFT-technique represent promising routes to improve the mechanical properties of nanotube based composites [18,19]. Because of attractive van-der-Waals forces, CNT tend to stay aggregated or in case of SWCNT bundled, whether or not treated by ultrasound. An impregnation of the CNT with MAO is necessary to achieve a satisfactory distribution of nanotubes in a polyolefin matrix [33]. Bonded or adsorbed (co-)catalyst, usually MAO, stabilizes the suspension of the dispersed nanotubes due to the repulsive forces between the MAO units, which in turn prevent the CNT from re-agglomerating.

The catalyst activity is not significantly influenced by the presence of (dried) nanotubes. The preparation of CNT containing nanocomposites depended on the same factors like the *in-situ* polymerization of neat polyolefins. The activity is influenced by the [Al]/[Zr] ratio, which in some cases depended on the pre-treatment. Of course, certain functional groups and impurities on the tube surface especially adsorbed water are able to hydrolyze MAO. Besides which, when using the pre-treatment method, where a covalent bonding is established between MAO and the filler, the number of functional groups on the CNT surface naturally control the [Al]/[Zr] ratio.

#### 2.2.1. Polyethylene Carbon Nanocomposites

Polyethylene multiwalled carbon nanotube (MWCNT) composites were prepared with filler contents of 0.6–3.3 wt%. Table 6 shows some experimental data of the *in-situ* polymerization. As metallocene, catalyst 3 ([(pMePh)_2_C(Cp)(2,7-bis-tBuFlu)]ZrCl_2_) was used.

The activity decreased with the amount of carbon nanotubes in the composite materials to a tenth of the original value while the molecular weight of the produced polyethylene increased from 700,000 to 1,050,000. The melting temperatures between neat and composite PE were nearly constant.

The incorporated amounts of CNT increased T_c_ of the resulted nanocomposites in comparison to neat PE. The reference PE had a T_c_ of about 117.6 °C; 1.0 wt% MWCNT led to an increase of about 8.7 K und a maximum was found at 1.6 wt% filler content, which caused an increase of 8.2 K. Even higher filler loadings led to lower T_c_s, but still leveled above that found for pure PE. For example a nanocomposite containing 18.0 wt% of CNT had a T_c_ of 115.4 °C. The half-time crystallization, representing the CNT nucleating effect, for all samples shown in Table 6 reached at 123 °C values of about 116 min. and is less than of polypropylene/MWCNT composites. The conductivity of some PE/CNT samples was evaluated using two-point measurements. Only samples with filler contents as high as 4 wt% show a strong increase in conductivity of 100,000 and reach 10^−6^ S/cm.

#### 2.2.2. Polypropylene Carbon Nanocomposites

Carbon nanofiber (nanowires) are interesting as fillers for polypropylene, because they represent an intermediate between traditional bulk carbon fibers of a large diameter and carbon nanotubes with a diameter in the range of a very few nanometers.

They are available in large quantities at low costs, which make them even more attractive [36]. In contrast to CNT the nanosized fibers are stiff and inflexible, their agglomerates are not so knotted and so they are comparably easy to separate by ultrasound.

Isotactic polypropylene (iPP) nanocomposites were prepared by *in-situ* polymerization using Cat2, and syndiotactic polypropylene (sPP) nanocomposites using Cat3.

**Figure 6.**
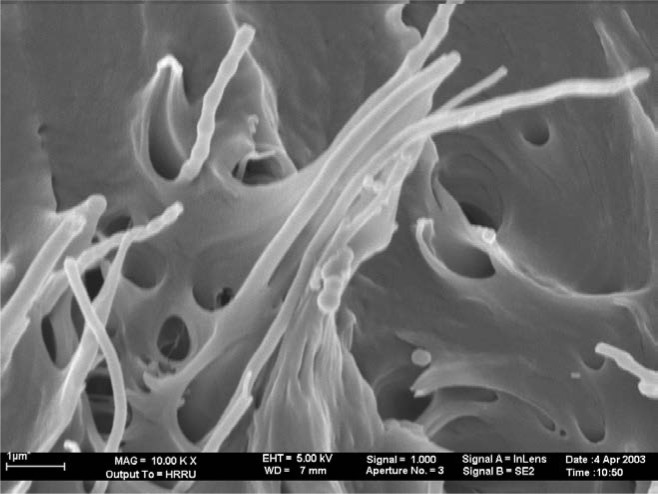
Syndiotactic polypropylene carbon nanofiber (CNF) composite material. The CNF has a diameter of 100 nm.

The morphology and the quality of the adhesion of the PP matrix to the CNF were obtained from SEM micrographs [37–39]. In Figure 6 it can be seen, that the wetting of the fibers by the polymer is good and the fibers were separated from each other, although they were loosely agglomerated. A coat of about 50 nm isotactic polypropylene covers the carbon nanofibers with a diameter of 100 nm.

Separated polymerization active sites at the surface of fibers form strings of pearl like structures. The polypropylene matrix draws on the fiber, showing the excellent adhesion between the hydrophobic polymer and the hydrophobic fibers. It can also be seen from this picture, that fiber pull-out has occurred to a certain degree which is obvious from the holes in the polymer matrix.

Incorporated carbon nanofiber led only to a slight increase (about 2 °C in comparison to neat sPP or iPP) of the composite melting point and largely unaffected the crystallinity, independently of the weight percentage. In contrast to that, the crystallization temperature and the halftime of crystallization were much more influenced by the presence of CNF and depended strongly on the quality of the filler distribution. For example a nanocomposite prepared by *in-situ* polymerization, which contains only 1.0 wt% CNF increased the crystallization temperature by 5 °C, while for the same improvement 7 wt% must be added when using melt compounding [37]. Possibly the *in-situ* polymerization, or the opportunity to accomplish the pre-reaction in a low viscosity solvent, led to a more homogenous CNF distribution. The values of the half-time of crystallization taken from isothermal DSC measurements showed a decrease of τ_1/2_ when carbon nanofibers were incorporated. In contrary to CNT a higher contents of nanofibers (range 0.1–1.1 wt%) did not seem to modify τ_1/2_ further. It was decreased to roughly one-third of the value for pure iPP and one-half for pure sPP in all.

**Figure 7.**
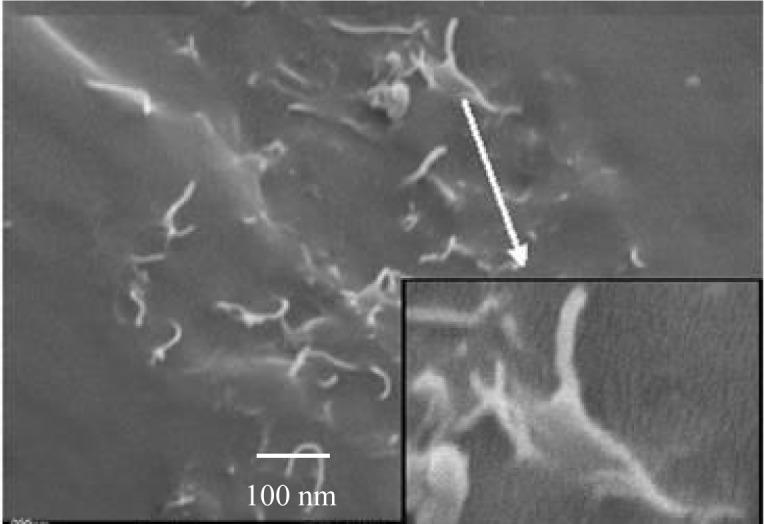
Isotactic polypropylene (PP) single walled CNT composite, Magnification 150,000×, for enlarged 400,000×.

The use of carbon nanotubes or multiwalled carbon nanotubes as filler increased the properties of the composite materials. The excellent incorporation of single walled CNT is shown in Figure 7.

All nanotubes are separated from each other. The iPP draws on the fiber as in the other cases. MWCNT are of more interest because they are much cheaper and have nearly the same properties.

**Figure 8.**
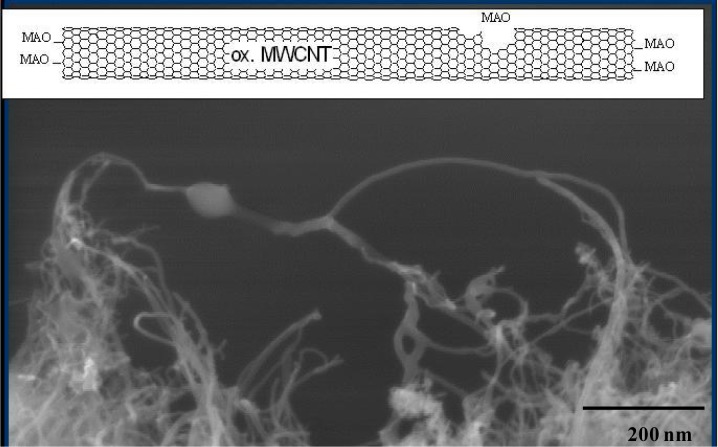
Transmission electron microscopy (TEM) micrograph of oxidized MWCNT with a ball of iPP at an active site after 20 min polymerization time, magnification 100,000×.

The morphology of the isotactic PP/MWCNT nanocomposites prepared by *in-situ* polymerization was investigated by using SEM or transmission electron microscopy (TEM). Figure 8 shows a SEM micrograph of iPP/CNT nanocomposite material. The few active sites on the surface of the nanotubes produce a ball of polymer, which grows on the fiber.

If the MWCNT are oxidized by nitric acid (ox. MWCNT) more defects are formed on the surface and more MAO can be absorbed resulting in more active polymerization sites. Using Cat1 ([Me_2_Si(2- Me-Ind)_2_]ZrCl_2_/MAO) an activity of 25,000 kg_Pol_/(mol_Zr_·h·mol_Mon_/L) is reached by a polymerization temperature of 30 °C, for Cat2 (see Figure 3) an activity of 55,000 kg_Pol_/(mol_Zr_·h·mol_Mon_/L). A dependence of the filler content on the polymerization activity was not found.

**Figure 9.**
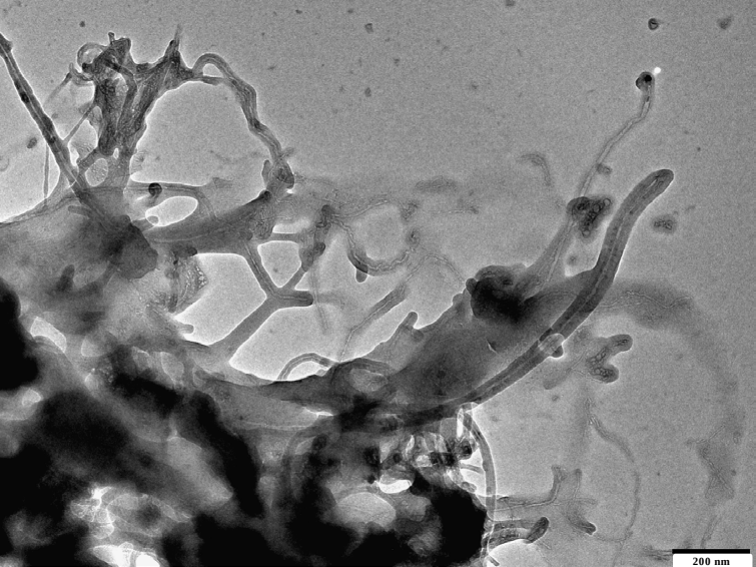
TEM image of polymer encapsulated ox. MWCNT. Almost every nanotube is covered by a thin and homogenous *in-situ* grown iPP film (about 10 nm).

Under these conditions the CNF are better covered by the polyolefin. It can be seen from Figure 9. that the nanotubes are coated by a thin film of iPP. The diameter of the MWCNT used (about 20 layers) is 20 nm and the thickness of the iPP coat about 8 nm. Even the loose network of polymer layered nanotubes on the lower left side, which are partially conglomerated by PP but still mostly separated from each other is permeated with polymer and seems to be widening with the growth of the polymer chains.

By longer polymerization times, the thickness of the polyolefin covering the fiber increased. The fiber/MAO/zirconocene system works like a supported catalyst. Filler contents between 0.5 and 50 wt% were possible.

CNT act as nucleating agents, therefore improvements on the crystallization behavior were expected. The crystallization temperature (T_c_), crystallinity (X_c_) and the half-time of crystallization (τ½) play an important role during the processing of these materials. By reduction of the cooling time required for part solidification, cycle times can be shortened. This can be accomplished by the incorporation of nucleating agents into the neat polymer which accelerate the crystallization.

The effect on the crystallization temperature was most emphatic. Pure iPP had a T_c_ of about 111 °C. The addition of 0.1 wt% MWCNT led to an increased T_c_ of 4–5 K. At higher filler contents the enhancement of the crystallization temperature rose rapidly to an amount of 9 K above pure iPP which was reached at a MWCNT content of 1.5–2 wt%. In the case of sPP/MWCNT composites the maximum enhancement of T_c_ was even up to 15 K higher in comparison to neat sPP, which has a crystallization temperature of about 95 °C. The half time of crystallization is the length of time needed for 50% of the crystallizable material to solidify from the melt. It depends on the (isothermal) crystallization temperature and on the filler content. The crystallization kinetic influences the morphology, which in turn affects the mechanical properties of a semi-crystalline polymer. The half-time of crystallization is reduced significantly with low amounts of nanotubes at all temperatures.

At 130 °C the half-time is 8 min for an iPP/MWCNT composite with 0.1 wt% filler, while 2.5 wt% reduced τ_1/2_ to only 1.5 min. When the percentage of MWCNT was raised to more than 3 wt% no further appreciable reduction of τ_1/2_ was obtained.

**Figure 10.**
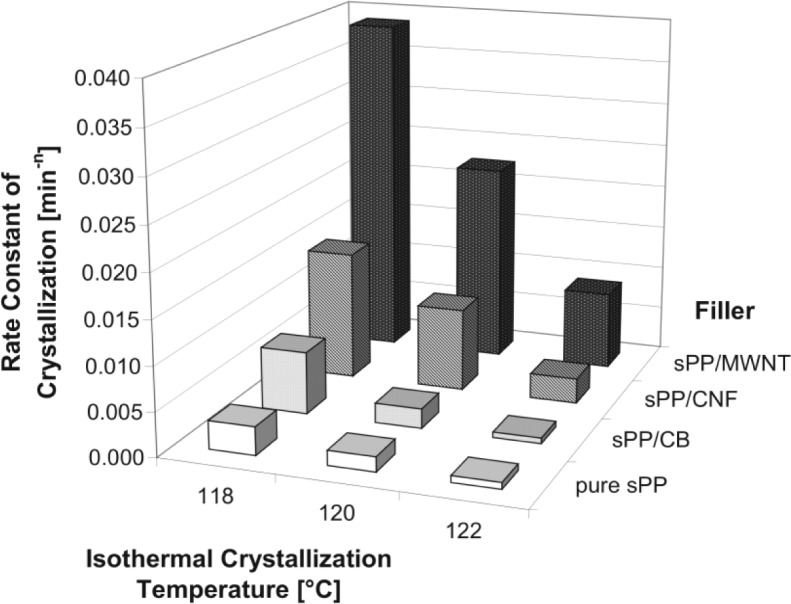
Crystallization rate in dependence of different syndiotactic PP composite materials and by three isothermal crystallization temperatures, CB = carbon black for comparision.

The effect can be seen by the crystallization rate at a constant temperature (Figure 10).

As expected, carbon nano tube filled composite materials show the highest crystallization rate followed by carbon nano fiber and CB filled syndiotactic polypropylenes. The value for sPP/MWCNT composite is about 20 times higher than for unfilled sPP.

The main advantage of CNF or MWCNT filled PP is the change of mechanical properties. High molecular weight isotactic polypropylene filled with MWCNT is an exceptionally strong composite material. The tensile strength of a composite film increases by 20% if only 1 wt% of MWCNT is incorporated but also the form stability and the crystallization rate from a melt increase strongly and make this composite material suitable for new applications such as in the automotive plastic industries.

Table 7 summerizes the polymerization activities and some polymer properties of isotactic.

**Figure 11.**
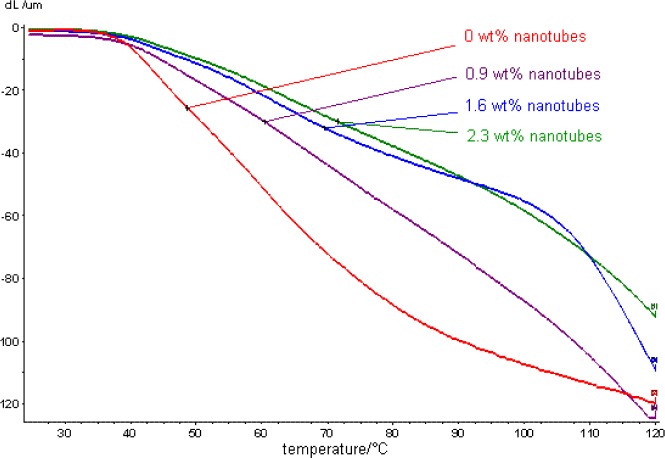
Dynamic mechanical measurements of iPP/MWCNT composites measuring the form stability in dependence of the filler content and temperature.

PP/MWCNT composites as well as the temperature form stability in dependence ot the filler amount. It can be seen, that there is nearly no dependence of the PP molecular weight and the melting temperature from the filling-grate. The polymerization activity of the homopolymerization is more than ten times higher than the activity in the presence of the nanotubes. Figure 11 shows the dynamic mechanical analysis of these iPP/MWCNT composites for measuring the form stability.

The declination by a constant force decreases with the temperature, but less for CNT filled polypropylene. The stiffness of a sample increased therefore with the amount of CNT in the composite.

The temperature at which a deflection of an iPP/MWCNT sample caused by a force is not reversible (temperature form stability) is of unfilled iPP 48.7 °C (Table 7). A composite PP with 0.9 wt% of MWCNT shows form stability up to 60.4 °C and a composite PP with 2.3 wt% of MWCNT a form stability of up to 71.5 °C. This shows, that only small amounts of carbon nanotubes can increase the form stability by more than 20°C. A form stability of 100 °C is desirable for most applications, which could be reached by a filler content of 6–8 wt%.

### 3. Experimental Section

All polymerizations were performed in dry toluene or dry heptane at 30–70 °C in a 1 L glass autoclave under inert gas atmosphere (argon). The first step of preparation was the absorbance of MAO onto the filler surface. The second step was the addition of a transition metal compound and the formation of catalytically active sites on the surface and the addition of ethene or propene. The monomer pressures were in the range of 2–10 bar.

Prior to the impregnation with MAO, the dried monospheres were dispersed in 20 mL of toluene and the desired amount of MAO-solution was added. The mixture was stirred for 24 h at room temperature. It was then filtered using a D4 fritted glass filter, washed with toluene and dried 4 h under vacuum. The *in-situ* polymerizations with silica monospheres/MAO were carried out in 200 ml toluene mixed with 2 mmol of TIBA as scavenger. The amount of M200/MAO was kept constant at 0.55 g, and the polymerization time was between 30 and 180 min. The catalyst *rac*-dimethylsilylbis(2-methyl- indenyl)zirconium dichloride (Cat1) (see Figure 1) was dissolved in toluene and led to a high isotactic polypropylene matrix with isotactic pentads of 95% ± 2%, typical for the catalyst. For the production of an (ultra) high molecular mass PP *rac*-dimethylsilylbis(2-methyl-4-(1- naphyl)indenyl)zirconium dichloride (Cat2) was used as the catalyst. The isotactic pentads were 97% ± 2% and the molecular mass was about 1,500,000 g/mol. The C_s_-symmetric metallocene, Di(p-methylphenyl)methyl(cyclopentadienyl(2,7-bis-tert-butylfluorenyl)) zirconium dichloride (Cat3) is used for sPP matrices.

Carbon nanofibers were dispersed in toluene solution, than MAO, an isotactic working zirconocene, and propene were added and the suspension stirred for approximately 30 min.

In the case of multi walled carbon nanotubes (MWCNT), they were sonicated in a toluene suspension, treated by MAO stirred for 24 h, filtered, and washed with hot toluene. After adding the chiral ansa zirconocene Cat2 and propene isotactic high molecular weight polypropylene iPP/MWCNT, composites with 0.9–50 wt% filler content were obtained. The molecular weights (Mw) of the polypropylene matrix in the nanocomposites were carried out by gel permeation chromatography using a Waters GPC 2000 Alliance system equipped with a refractive index detector, viscosimetric detector and a set of three colums, Styragel type. The solvent used was 1,2,4-trichlorobenzene. The analyses were performed at 140 °C and 1.0 mL/min. The molecular weights were in the range of M_w_ = 1,200,000–1,700,000. The polymerization activity reached 5000 kg_PP_/mol_Zr_·h [propene].

## 4. Conclusions

Polyolefin nanocomposites open up an approach to new classes of materials with particularly good property combinations. A soft polyolefin matrix can be combined with hard inorganic particles or strong layers of silicates or graphene or with fibers of extreme high tensile strength. Carbon nanofibers (CNF) or multiwalled carbon nanotubes (MWCNT) are an especially attractive class of fillers for polymers because of their intriguing mechanical and thermal properties. It could be shown, that an easy way to prepare such polyolefin nanocomposites is the *in-situ* polymerization using nanoparticles such as silica balls or carbon nanotubes activated by metallocene/MAO or other single site catalysts. Particularly when soluble single site catalysts are used for *in-situ* polymerization, the separation and distribution of the nano-sized particles or fibres in the polyolefin matrix is very good. Materials can be obtained with high gas barrier resistance, high thermal and electric conductivity, high form stability, and a significant flame redardancy as well as other excellent properties. There is great hope, that graphene filled polyolefins can achieve most of these advantages simultaneously [40,41]. Our investigations have shown, that even low parts (2–4 wt%) of carbon nanotubes in the composite material, highly dispersed by an *in-situ* polymerization technique increased the form and temperature stability by 30%–100%. The better stiffness and the temperature form stability of polyolefin carbon-nanotube or nanofiber composites by high aspect ratio make such materials suitable in the near future for many applications, such as in the automotive industries, using simple injection molding processes.

## Figures and Tables

**Table 1.** Polyethylene nanocomposites prepared with different inorganic nanoparticles and different parts of filler by *in-situ* polymerization with metallocene catalysts.

Filler	Activity (kg_PP_/(mol_Zr_·h·mol_Mon_/L))	Part of Filler (wt%)	Molecular weight (Mw: g/mol)	Melting point (°C)
SiC	197,000	1.46	196,000	142
TiC	243,000	1.59	195,000	142
CdSe	151,200	1.58	180,000	138
CdSe	57,000	9.62	65,000	133

Polymerization conditions: 5 × 10^−8^ mol Cat1, 160 mg MAO, 5.5 bar ethene pressure, 200 mL toluene as solvent, 60 °C polymerization temperature, 30 min reaction time.

**Table 2.** Polypropylene nanocomposites of different inorganic nanoparticles and different parts of filler by *in-situ* polymerization with metallocene catalysts.

Filler	Activity (kg_PP_/(mol_Zr_·h·mol_Mon_/L))	Part of Filler (wt%)	Molecular weight (Mw: g/mol)	Melting point (°C)
SiO_2_	11,600	1.69	179,000	159
CaCO_3_	17,600	1.48	114,000	157
Al_2_O_3_	14,200	1.85	259,000	160
CoFe	5,300	1.21	254,000	160
BN	18,500	1.38	259,000	160
MgO	13,800	1.47	175,000	157
MgO	3,900	9.22	242,000	159

polymerization conditions: 1.3 × 10^−6^ mol Cat2, 400 mg MAO, 1 bar propene pressure, 400 mL toluene as solvent, 60 °C polymerization temperature, 4 h reaction time.

**Table 3.** Polymerization activity and polymer properties in relation to polymerization temperature, propene concentration, and catalyst amount.

Polymerization Temperature (°C)	Propene Concentration (mol/L)	Catalyst Amount (mol)	Activity (kg_Pol_/(mol_Zr_·h·mol_Mon_/L))	Melting Point (°C)	Molecular Weight (Mw) (g/mol)
0	1.4	1.30 × 10^−6^	600	149	790,000

	0.6	1.40 × 10^−6^	1,800	137	370,000
30	1.4	1.30 × 10^−6^	2,300	141	560,000
	3.5	6.00 × 10^−7^	2,500	142	640,000

60	1.4	7.00 × 10^−7^	3,000	121	220,000

Polymerization conditions: polymerization time (30–180 min, solvent 200 mL of toluene, amount of M200/MAO 0.55 g, amount of triisobutylaluminium (TIBA) 2 mmol, metallocene: Cat3.

**Table 4.** Stability of MAO impregnated nano silica balls in syndiotactic propene polymerization.

Weeks after preparation	Activity (kg_PP_/(mol_Zr_·h·c_Mon_))	Filler content (wt%)
0	3700	11
2	3300	9
11	3000	10

Polymerization conditions: polymerization temperature 30 °C; polymerization time 30 min; 2 bar propene pressure; solvent 200 mL toluene; 0.55 g silica/MAO; zirconocene = 1.3 × 10^−6^ mol/L; TIBA 2 mmol.

**Table 6.** Polymerization activity and polymer properties of polyethylene/multiwalled carbon nanotube (MWCNT) composites.

Amount MWCNT (mg)	Filling-grade (wt%)	Activity (kg_PE_/(mol_Zr_·h·c_Mon_))	Molecular weight (Mv) (g/mol)	Melting temperature (°C)	Crystallization temperature (Tc) (°C)
0	0	2.7 × 10^4^	700,000	139	107.6
18	1.0	5.8 × 10^3^	818,000	139.8	116.3
42	1.6	6.9 × 10^3^	790,000	138.3	116.8
100	1.6	5.4 × 10^3^	1,000,000	138.7	116.5
190	2.1	4.0 × 10^3^	1,050,000	137.4	116.6

Polymerization time: 30–120 min; solvent: 200 mL toluene; MAO: 300 mg; zirconocene: 2 × 10^−6^ mol Cat3 ([(pMePh)_2_C(Cp)(2,7-bis-tBuFlu)]ZrCl_2_); Mv: viscosity average molecular weight.

**Table 7.** Polymerization activities, viscosity average molecular weights, melting temperatures, and form stability temperatures of iPP/MWCNT samples with different amounts of fillers at a force of 1.8 MPa.

Amount MWCNT (mg)	Filling-grade (wt%)	Activity (kg_pp_/(mol_Zr_·h·c_Mon_))	Molecular weight (Mv) (g/mol)	Melting temperature (°C)	Temperature form stability (°C)
0	0	5.6 × 10^4^	1,800,000	160.2	48.7
50	0.9	4.3 × 10^3^	1,900,000	161.3	60.4
90	1.6	3.8 × 10^3^	1,800,000	160.9	69.5
103	2.3	4.6 × 10^3^	2,400,000	161.0	71.5

Polymerization time: 15–30 min; solvent: 200 mL toluene; MAO: 200 mg; zirconocene: 2 × 10^−6^ mol Cat2.
